# The Seroprevalence of *Chlamydia abortus* in Montana Domestic Rangeland Sheep Varies by Breed, Flock and Herding Practices

**DOI:** 10.1002/vms3.70935

**Published:** 2026-04-03

**Authors:** Avia J. Simmons, Joanna‐Lynn C. Borgogna, Brent L. Roeder, Carl J. Yeoman, Christian J. Posbergh

**Affiliations:** ^1^ Department of Microbiology & Cell Biology Montana State University Bozeman Montana USA; ^2^ Weissman Hood Institute Touro University Great Falls Montana USA; ^3^ Touro College of Osteopathic Medicine Montana Great Falls Montana USA; ^4^ Department of Animal & Range Sciences Montana State University Bozeman Montana USA

**Keywords:** *Chlamydia*, Montana, seroepidemiologic study, sheep

## Abstract

**Background:**

Ovine enzootic abortion (OEA) is a reproductive disease in domestic sheep characterized by late‐term abortions occurring 2–3 weeks before term and leading to economic losses for sheep producers. OEA is caused by *Chlamydia abortus*, an obligate intracellular bacterium, which is primarily spread through intranasal transmission from infected aborted fetal contents and vaginal discharge. This method of transmission leads to rapid transmission within and between flocks each lambing season, with infected ewes aborting in the subsequent lambing season.

**Objectives:**

While there are surveys performed in other countries to determine the seroprevalence of *C. abortus* in domestic sheep populations, very few have been performed in the United States. We performed the first seroprevalence survey on *C. abortus* in Montana domestic rangeland sheep.

**Methods:**

We collected serum samples from 781 domestic sheep from 17 flocks in Montana, which were used to determine the seroprevalence through indirect ELISA.

**Results and Conclusions:**

In total, 95 (12.6%) of the sheep tested had *C. abortus* anti‐major outer membrane protein (anti‐MOMP) antibodies. Seropositivity varied significantly by breed, flock, whether the animals were herded and whether the tested ewe had already lambed that season prior to sampling. These data provide insight into the impacts of OEA in Montana domestic rangeland sheep and will better inform local producers about whether additional treatments and preventative measures should be put in place.

## Graphical abstract

Ovine enzootic abortion, caused by *Chlamydia abortus*, leads to significant reproductive losses in sheep. This study found 12.6% of 781 sheep in Montana tested positive for antibodies with differences by breed, flock and herding status.

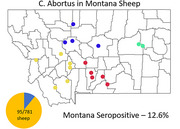

## 1 Introduction

Ovine enzootic abortion (OEA) significantly affects domestic sheep populations worldwide. OEA is characterized by the late‐term abortion of ewes within the 2–3 weeks prior to term birth or birth of lambs that fail to thrive. OEA is caused by *Chlamydia abortus*, a non‐motile, obligate intracellular bacterium characterized by a unique biphasic lifecycle within a host where infectious, non‐metabolically active elementary bodies (EBs) enter the host cell, replicate as metabolically‐active reticulate bodies (RBs), then the host cell lyses, wherein the RBs transform into EBs to infect other cells (Hackstadt et al. 1997; Rodolakis and Laroucau 2015). The most common form of transmission between sheep is inhalation of *C. abortus* present on aborted fetal tissue, placenta, and vaginal fluids from an infected ewe (Wilsmore et al. 1984). Therefore, most infection events take place during lambing seasons (Aitken and Longbottom 2007; Blewett et al. 1982). Other transmission routes are possible but are not well‐studied and considered rare (Wilsmore et al. 1984). Once infected, the ewe will be largely asymptomatic until the next lambing season, when they will then abort and develop a life‐long low‐level immunity, though they can still spread *C. abortus* to other sheep (Rodolakis and Souriau 1980; Stamp et al. 1950). In around 10% of infections, ewes will abort multiple times, potentially becoming permanently infertile (Rodolakis and Souriau 1980).

Within the United States, there are very few reports on the prevalence of *C. abortus* in domestic rangeland sheep populations. Within the Intermountain Western United States, *C. abortus* accounted for approximately 15% of abortions in a 2017 study (Wilson et al. 2017). In other countries, *C. abortus* has been found to have a seroprevalence between 4% and 27% within domestic sheep (Turin et al. 2022). *C. abortus* infections also lead to an annual cost of £16.5 million in the United Kingdom (Bennett and Ijpelaar 2005), with each ewe that aborts costing over £200 (Robertson et al. 2018). These screenings are largely absent within the United States, making the overall impact of *C. abortus* on sheep flocks difficult to discern. Other methods include testing ewes and aborted fetal tissues post‐abortion; these samples may be difficult to collect and often too late to prevent the spread to other ewes within the flock (Longbottom and Coulter 2003; Gebretensay et al. 2019; Turin et al. 2022). Without knowledge of the prevalence of *C. abortus* and its lifecycle within hosts, it is difficult to implement additional management strategies that may increase profitability and animal health.

Sheep within Montana are primarily concentrated on extensive rangeland operations that run 100 or more breeding ewes at a time and are typically composed of fine‐wool breeds such as Targhee, Rambouillet or related crossbreeds. Larger flocks may employ herders to manage the flock during summer grazing on native rangelands, ensuring optimal health and production. Montana sheep are primarily raised for meat and wool, and some flocks choose to retain breeding stock for regional and national marketing to other sheep producers.

Here, we partnered with 17 different domestic rangeland sheep producers in the state of Montana. Varying flock sizes, management strategies, breeds and age ranges of operations were reflected in our study population, which represented the sheep population in Montana. We collected serum from 781 sheep to measure the presence of antibodies against *C. abortus*. Rather than assessing the presence of active infections, we sought to estimate the seroprevalence of *C. abortus* antibodies within the domestic sheep population. In addition, all sheep sampled had been vaccinated against *C. abortus* in the past, which can produce antibodies that are occasionally indistinguishable from natural infection. From this information, we determined whether different management strategies and the breed, age and sex of their sheep impacted the seroprevalence of *C. abortus* within their flocks.

## 2 Methods

### 2.1 Sample Collection

Blood was collected from the jugular vein through a clean 21G needle into Vacutainer serum tubes (Becton, Dickinson and Company, NJ, USA), allowed to coagulate for 30 min at room temperature, then placed on ice to transport to the Animal Biosciences Laboratory at Montana State University (45° 40′ 09.5″ N, 111° 03″ 11.3″ W). The blood was centrifuged at 2000 rpm for 15 min; serum was extracted by pipette and placed into 1.5 mL microcentrifuge tubes and placed at −80°C until analyzed. Samples from 781 sheep across 17 flocks were collected. Around 16 of the 17 total flock sites had samples collected during the 2024 lambing season (March–May). The final site was collected in August 2024 (*n* = 50). Sheep producers across the state were invited to participate in the study, among those that were willing to participate, 17 flocks that most broadly represented the sheep‐rearing regions of the state were selected as a representative sample of the state. Each producer is labelled with a number 1–17 to maintain anonymity and de‐identify flocks. Within each flock, animals were randomly selected. At the time of sampling, each animal's sex, age (when known), breed (as identified by each producer), lambing and abortion statuses for the season, and any known health issues were recorded. Each flock was also identified as to whether they raised rams for breeding stock, if the flocks are herded throughout the summer or left alone in a pasture, and whether they lamb intensively, where there is more confined feeding of lambs and ewes, lambs wean at a younger age and a higher lambing rate is expected.

### 2.2 Indirect Enzyme‐Linked Immunosorbent Assays

Sera were tested for the presence of *C. abortus* antibodies using the ID Screen *Chlamydophila abortus* Indirect Multi‐Species ELISA kit (Innovative Diagnostics Vet, Grabels, France) according to manufacturer instructions. Each plate was read on a BioTek Epoch2 microplate spectrophotometer at 450 nm to determine optical density (OD) (BioTek Instruments, VT, USA). Identification of seropositive and seronegative samples was based on the formula provided by the manufacturer: (OD_sample_ / OD_positive control_) × 100, with a value greater than 60 defined as seropositive, a value less than 50 defined as seronegative, and values between 50 and 60 being inconclusive. Each sample was tested in triplicate. Thirteen samples were initially defined as inconclusive (each of the three tests were inconclusive), but subsequent retesting and averaging across each test classified them as seropositive or seronegative.

### 2.3 Statistical Analysis

Pearson's *χ* ^2^‐tests were conducted against each sample characteristic and their resulting seroprevalence (Plackett 1983). In cases of only one degree of freedom present in a *χ* ^2^‐test, a Yates continuity correction was used (Adler 1951). Pairwise logistic regression was performed between each sample characteristic to determine significant differences in seroprevalence of each group. Age was grouped into young (1–2 years) and mature (>2 years) for additional analyses, with samples lacking age data (*n* = 240) being excluded from age‐related analyses. For flock‐level variables, a flock was determined to be seropositive if at least one sheep within the flock was seropositive. Flock‐level variables were analyzed by mixed effects logistic regression models to account for within‐flock biases. For each statistical test, a Benjamini–Hochberg correction was used to control the false discovery rate (Benjamini and Hochberg 1995). A final multivariate mixed effects logistic model was developed based on significant *p*‐values (adjusted *p*‐value < 0.05) in the corresponding univariate logistic regressions and *χ* ^2^‐tests with producer as the random effect using the glmer function in the *lme4* package (version 1.1‐35.5) in R Statistical Software. The model assumed a binomial distribution with a logit link function. *p*‐value estimation from this function uses the Satterthwaithe approximation for degrees of freedom (Satterthwaite 1946), with each fixed effect *p*‐value estimated using the asymptotic Wald test. The *geosphere* package (version 1.5‐18) in R Statistical software was used to perform hierarchical distance clustering for each flock's GPS coordinate (Figure 2A). Clusters were determined based on centroid‐linkage clustering, with the cutoff point defined as the midpoint on the dendrogram tree with the maximum distance between the merging point. These clusters were also analyzed using Pearson's *χ* ^2^‐test and pairwise logistic regression. Other R Statistical Software packages used include *MASS* (version 7.3‐61) for conducting the *χ* ^2^‐tests and *ggplot2* (version 3.5.1) for visualization.

## 3 Results

### 3.1 Flock Characteristics

Of the 17 flocks where samples were collected, six raise rams for breeding stock, seven have herded sheep and five are classified as intensive lambing operations (Table 1). Study flocks had a range of ewe numbers from 100 to 4000, however we are not disclosing specific flock size for each sampled flock to preserve confidentiality of participating producers. A total of 740 ewes and 41 rams were sampled from throughout the state of Montana for this study. Fifteen breeds were represented, and ranged in age from 1 to 11 years (Table 1). However, only six breeds (Polypay, Rambouillet, Targhee, Targhee‐Rambouillet, Texel cross and white‐faced crossbred) had enough representation for statistical analysis by breed; other breeds were excluded from breed‐specific analyses. Separate analyses were also performed by grouping the crossbreeds (commercial, crossbred, Targhee‐Rambouillet, Texel cross and white‐face crossbred) into a single ‘crossbreed’ group.

**TABLE 1:** Sample characteristics.

	Total *n*	Seropositive *n* (Percent)
**Breed**		
Columbia	1	0 (0)
Commercial	15	3 (20)
Crossbred	3	2 (66.67)
East Friesian	8	2 (25)
Hampshire	1	0 (0)
High Line Rambouillet	1	0 (0)
Old Rambouillet	1	1 (100)
Polypay	50	2 (4)
Rambouillet	300	42 (14)
SAMM	4	1 (25)
Suffolks	3	0 (0)
Targhee	245	22 (8.98)
Targhee‐Rambouillet	50	7 (14)
Texel Cross	51	11 (21.57)
White‐Faced Crossbred	46	1 (2.17)
**Sex**		
Female	740	91 (12.30)
Male	41	4 (9.76)
**Age**		
Young (1–2 years)	154	13 (8.44)
Mature (>2 years)	397	41 (10.33)
**Producer Number**		
1	60	7 (11.67)
2	51	9 (17.65)
3	16	0 (0)
4	52	12 (23.08)
5	60	13 (21.67)
6	50	9 (18)
7	49	1 (2.04)
8	60	2 (3.33)
9	13	1 (7.69)
10	50	7 (14)
11	50	5 (10)
12	50	8 (16)
13	15	0 (0)
14	50	12 (24)
15	59	6 (10.17)
16	46	1 (2.17)
17	50	2 (4)
**Producer herds sheep?**		
Yes	363	57 (15.70)
No	418	38 (9.09)
**Producer raises rams for breeding?**		
Yes	312	44 (14.10)
No	469	51 (10.87)
**Producer lambs intensively?**		
Yes	230	22 (9.57)
No	551	73 (13.25)
**Lambed before sampling?**		
Yes	285	47 (16.49)
No	405	43 (10.62)
**Aborted before sampling?**		
Yes	3	0 (0)
No	687	90 (13.10)

### 3.2 Seroprevalence by Flock Characteristics

In total, 95 (12.16%) of the 781 tested sheep had *C. abortus* antibodies present. Of the 17 total flocks, only two sites did not have any seropositive sheep. This may be in part due to the lower sample numbers collected from both sites (*n* = 16 and 15; Table 1 and Supporting Information). Interestingly, of the three ewes tested that had aborted before sampling, none of them had *C. abortus* antibodies present and were all in different flocks. However, due to the low sample size for ewes that aborted prior to sampling (*n* = 3), this result should be approached cautiously.

**FIGURE 1:**
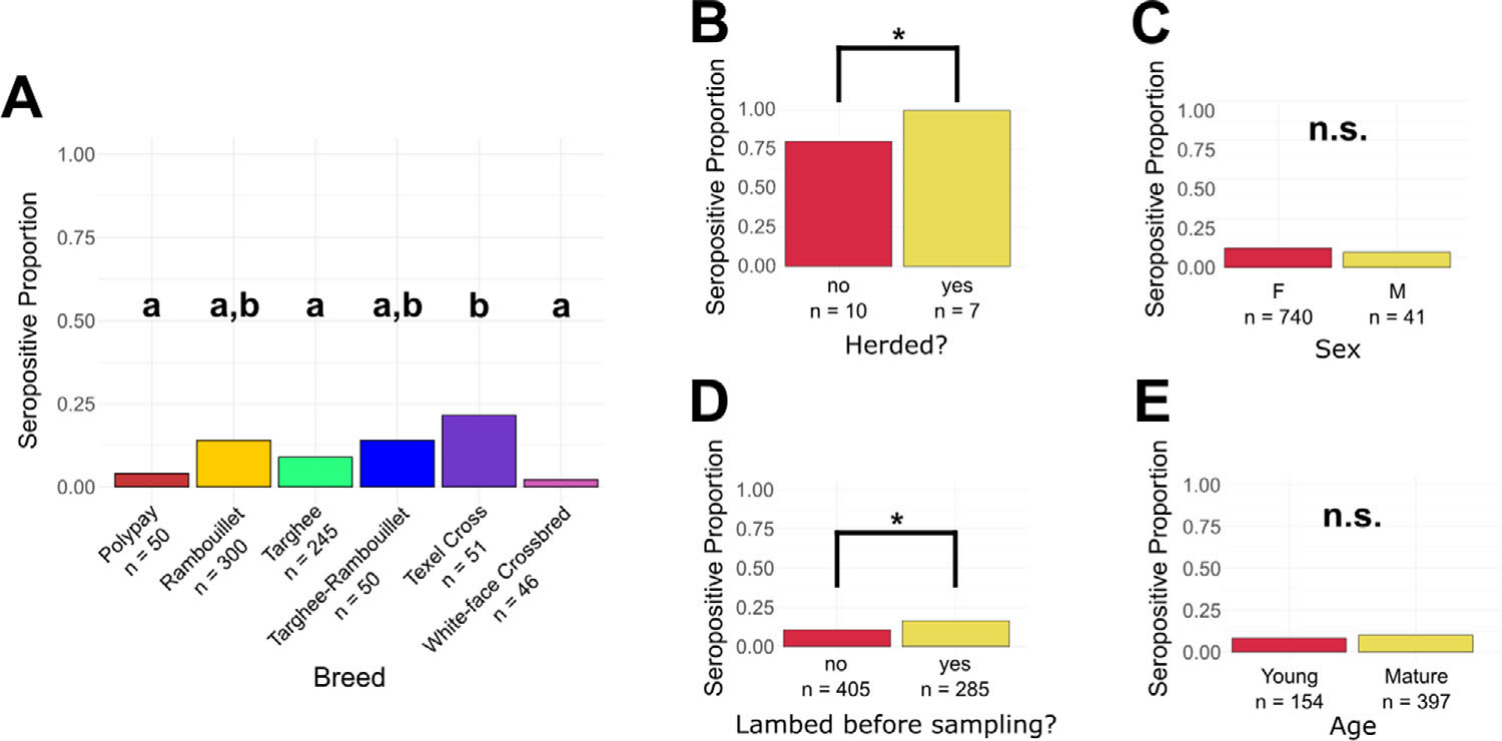
Seroprevalence by sample characteristics. Seroprevalence is broken down by breed (A), herded flocks (B), the sex of the animal (C), whether the ewe lambed before sampling (D), and age (E). The number of samples in each group is noted below each group's name. Herded flocks (B) are a flock‐level variable, with the other variables being animal‐level (A, C–E). Statistically significant pairs (adjusted *p*‐value < 0.05) are denoted by letter groups or an asterisk (*). Breed contains only groups with enough members for statistically significant comparisons.

**FIGURE 2:**
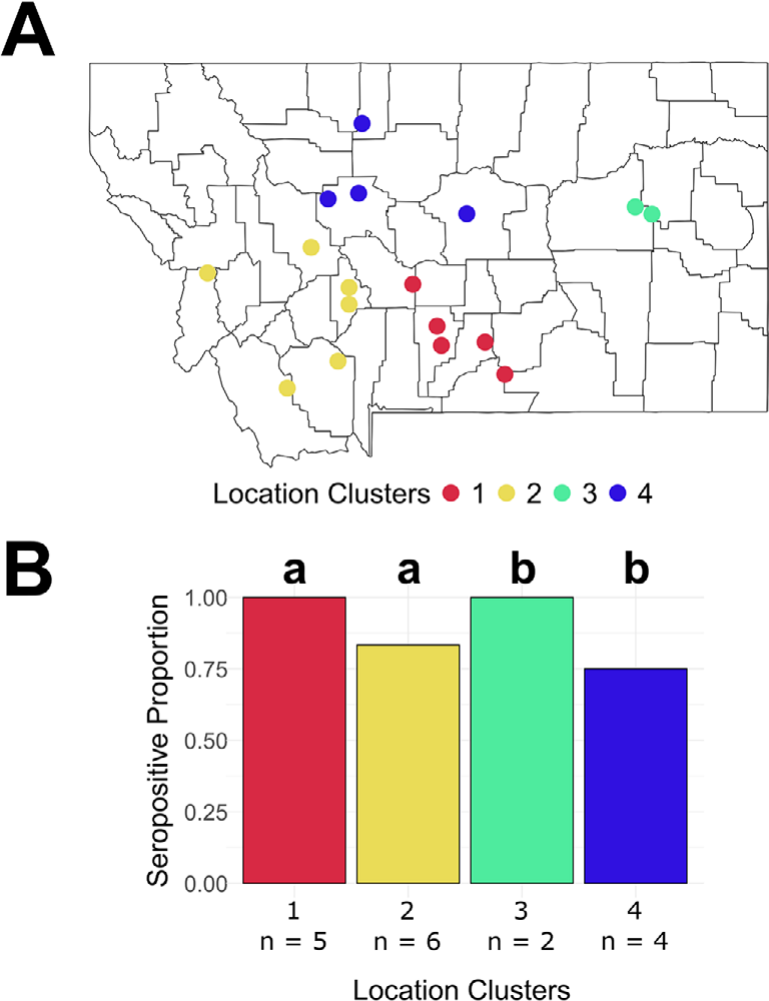
Seroprevalence by location clustering of producers. The location clusters (A) were determined by hierarchical clustering. The seroprevalence of each location cluster (B) has statistically significant pairs (adjusted *p*‐value < 0.05) denoted by letter groupings. The number of samples in each group is noted below each group's name.

Seroprevalence was found to differ significantly by breed, flock, location cluster and whether the sheep were herded based on both Pearson's *χ* ^2^ tests (Table 2) and logistic regression (Figure 1). Commercial (20%), East Friesian (25%) and texel crosses (21.6%) had higher seroprevalence compared to other breeds (Figure 1A). However, when grouping crossbreeds into one group, the seroprevalence did not differ significantly by breed (adjusted *p*‐value > 0.05). Herded sheep operations had a higher seroprevalence than non‐herded operations (Figure 1B). Seropositivity rates based on whether the ewe had lambed that season at time of sampling was also significant by logistic regression, but not by Pearson's *χ* ^2^ test, with ewes that already lambed being more likely to be seropositive (Figure 1D). With regards to location clustering, seroprevalence was greater in southern Montana compared to northern Montana (Figure 2). Seroprevalence was not significantly different by sex, age, intensive lambing operations and whether the ewe had aborted that season (Figure 1). When looking at the impact of interactions between sample characteristics on seroprevalence, breed and lambing status had a significant association, specifically between Targhee and Rambouillet sheep (adjusted *p*‐value = 0.013), whereas the two breeds were not significantly different from each other without interaction. When combining the crossbreeds, this interaction is still present. No other interactions yielded significant results. The final multivariate model was developed using two significant variables (lambed this season and location clusters), with the producer acting as a continuous variable. Breed and whether an operation herded sheep were excluded from the final multivariate model as they were not determined to have a significant effect on seroprevalence when accounting for other confounding variables (Supporting Information). Whether each ewe had lambed before sampling (adjusted *p*‐value = 0.00657) and clusters by location (adjusted *p*‐value = 0.00032) were determined to have a significant effect on *C. abortus* seroprevalence when accounting for producer (Table 2).

**TABLE 2:** *χ* ^2^‐test results for each sample characteristic. The Pearson's *χ* ^2^‐tests used a Yates’ continuity correction for variables with 1 degree of freedom.

Sample Characteristic	Pearson's chi‐square			
	*χ* ^2^‐statistic	df	*p*‐value	Corrected *p*‐value
**Animal‐level variables**				
Sex	0.057187	1	0.811	0.92685714
Age	0.25857	1	0.6111	0.77550769
Breed	17.548	7	0.01419	0.04540800*
Breed (crossbreeds combined)	8.5855	4	0.07225	0.14450000
Lambed	4.5841	1	0.03227	0.08605333
Aborted	2.4477 × 10^−30^	1	1	1.00000000
Producer	42.783	16	0.0003013	0.00482080*
**Flock‐level variables**				
Intensive	1.7302	1	0.1884	0.33493333
Raises rams	1.538	1	0.2149	0.34384000
Herded	7.3419	1	0.006736	0.02694400*
Location cluster	16.166	3	0.001048	0.00838400*

* indicates signficance at the P < 0.05 level.

## 4 Discussion

OEA is a biologically and economically harmful disease to domestic rangeland sheep populations. An infected ewe will lead to a minimum of a season's loss in lamb production, with a potential to abort for multiple seasons or to become permanently infertile (Rodolakis and Souriau 1980; Rodolakis and Laroucau 2015; Caspe et al. 2023). These ewes also have the potential to infect approximately a third of a naïve flock in a single lambing season, which may not be caught until the following lambing season (Rodolakis and Souriau 1980; Hackstadt et al. 1997). In the case of infected rams, each could potentially infect multiple ewes through both mating and import of their seminal fluid to other sheep operations (Teankum et al. 2007).

Most studies into the seroprevalence of *C. abortus* only investigate its presence in ewes (Teankum et al. 2007). Most domestic sheep operations rely on having significantly fewer rams than ewes, both for increased economic production, simplifying management strategies and to prevent fighting amongst rams (Maquivar et al. 2021; Shreffler and Hohenboken 1974), leading to fewer rams available for testing in general. This leads to a bias in the current literature; however, because the impact of *C. abortus* on the male population is largely unexplored. While one study demonstrated *C. abortus* to be present in seminal fluid (Teankum et al. 2007), it is unclear whether rams display any other clinical signs outside of occasional infertility. Whether rams can spread *C. abortus* between ewes during breeding also remains unexplored but could prove to be a transmission mechanism in vaccinated flocks. Our study did not demonstrate a difference in seroprevalence between rams and ewes, which highlights the importance of understanding disease pathology within the ram. Seroprevalence did not significantly vary by age, but a significant number of samples were excluded due to lack of age data, so analysis by age may have introduced bias.

Significant differences in seroprevalence occurred between flocks. This is likely due to many factors, including flock size, breed composition, climate and vaccination schedule. This may also be in part due to other management practices, such as having sheep herded or having shed versus pasture lambing. Herded flocks tend to be larger, thus increasing the chances for transmission of communicable diseases, highlighting the need for preventative management strategies in these environments to decrease exposure. All the flocks in this study shed lambed, where ewes lamb in smaller confined spaces to have better access to care and the ability to nurse and raise their lambs, due to poor weather during lambing season in Montana. Management strategies to prevent the spread of *C. abortus* to humans, such as limiting pregnant women's exposure to sheep during lambing and wearing personal protective equipment in known cases of infection, are known to help prevent human exposure (Turin et al. 2022). To prevent *C. abortus* spread between ewes during lambing, producers should keep ewes that abort separate from the main population or sell them and remove any aborted materials, in addition to keeping a standard of cleanliness within lambing pens if they are used (Longbottom and Coulter 2003; Aitken and Longbottom 2007). Vaccination is also a common management strategy to prevent disease spread that is generally effective, but many ewes will still shed *C. abortus* after being vaccinated (Caspe et al. 2020; Murcia‐Belmonte et al. 2019), especially if only given to a portion of the population.

Different sheep breeds in our study were found to have different seropositivity rates. Most flocks in our study were mostly comprised of 1–2 different breeds, which might be influenced by the flocks’ location, rather than by breed exclusively, though this interaction was not seen in this study. Differences in seroprevalence by location could also be driven by breed‐based factors, flock density, flock interactions and interactions with the environment (Nogarol et al. 2024). Other publications have reported correlations between breed and seroprevalence, with crossbreeds having a higher risk of being seropositive (Nogarol et al. 2024; Gebretensay et al. 2019), which is reflected in our data regarding commercial sheep, as the reported commercial sheep are crossbreeds. Other publications report no difference between breeds; suggesting additional cofactors might affect seroprevalence rather than solely breed susceptibility (McCauley et al. 2010). When developing the multivariate analysis, breed was excluded due to a lack of significance; however, there may be other confounding variables that influence this.

Commercial ELISA testing was used to determine seroprevalence in this study, which measures for the presence of antibodies against the *C. abortus* MOMP in sheep sera. This test cannot determine whether the presence of these antibodies is due to an active infection or from prior vaccination, as many commercially available vaccines lead to the production of antibodies that can lead to a false positive on ELISA tests (O'Neill et al. 2018). As all sheep in this study were vaccinated, the possibility of vaccine‐induced antibody production leading to false positives should be addressed in future studies through molecular techniques such as PCR or complement fixation to detect active infections, especially in placental tissues (Hireche et al. 2016).

*C. abortus* poses many difficulties in preventative screenings and treatment options. While it responds well to tetracyclines (Longbottom and Coulter 2003; Greig et al. 1982), given its lack of symptomatic activity within the host species, it can be difficult for producers to discern whether treatment is necessary, in addition to complications from restrictions on preventative antibiotic usage in sheep (Dessai et al. 2024). In many flocks, the most effective strategy is to vaccinate incoming rams and ewes, then re‐vaccinate as their vaccine schedule allows, either annually for inactivated vaccines or every 2–3 years for live‐attenuated vaccines (Rodolakis 1983). All sheep in this study were vaccinated against *C. abortus*, which implies the possibility of the bacteria remaining endemic despite vaccination efforts. This could potentially be due to some vaccines, such as the 1B strain, being able to produce an active infection in some ewes (Caspe et al. 2020), or from residual infection from a sold ram or ewe passing into another flock to infect other sheep in the subsequent breeding and lambing seasons (Longbottom et al. 2013).

## 5 Conclusion

This study was the first to determine the seroprevalence of *C. abortus* in rangeland domestic sheep in Montana. We found that *C. abortus* antibodies are present in a proportion of sheep despite routine vaccination efforts. However, with the knowledge we now have about this seroprevalence across sheep flocks, producers will be able to better determine whether their flocks are at greater risk. This study highlights the potential need for modification to vaccination schedules or other management practices to limit disease spread. The presence of *C. abortus* within the Montana domestic population poses risks not only for the health of the producer's flocks, but also for their continued economic production (Robertson et al. 2018). Therefore, looking into improved management strategies and understanding the process of *C. abortus* causing abortions in subsequent lambing seasons could improve domestic sheep production.

## Author Contributions

**Avia J. Simmons**: writing – original draft, writing – review and editing, visualization, formal analysis, conceptualization, investigation, data curation, validation, software, funding acquisition, methodology. **Joanna‐Lynn C. Borgogna**: writing – review and editing, conceptualization, formal analysis, investigation, funding acquisition, methodology. **Brent L. Roeder**: writing – review and editing, funding acquisition, conceptualization, investigation, methodology. **Carl J. Yeoman**: writing – review and editing, funding acquisition, project administration, resources, supervision, conceptualization, investigation, methodology. **Christian J. Posbergh**: writing – review and editing, funding acquisition, conceptualization, project administration, supervision, investigation, methodology.

## Funding

This research was funded by the Bair Ranch Foundation.

## Ethics Statement

All animal involvement and procedures were approved by the Montana State University Agricultural Animal Care and Use Committee (Protocol # 2024‐325‐AA) and by the Montana State University Institutional Biosafety Committee (Protocol # 2024‐490‐IBC).

## Conflicts of Interest

The authors declare no conflicts of interest.

## Supporting information




**Supporting Information**: vms370935‐sup‐0001‐Data.xlsx

**Supporting Information**: vms370935‐sup‐0002‐SuppMat.xlsx

## Data Availability

Sample collection data is included in supplemental materials.
